# FGF2 activates TRPC and Ca^2+^ signaling leading to satellite cell activation

**DOI:** 10.3389/fphys.2014.00038

**Published:** 2014-02-11

**Authors:** Yewei Liu, Martin F. Schneider

**Affiliations:** Department of Biochemistry and Molecular Biology, University of Maryland School of MedicineBaltimore, MD, USA

**Keywords:** TRPC, FGF2, satellite cell, calcium, NFATc3, NFATc2

## Abstract

Satellite cells, as stem cells of adult skeletal muscle, are tightly associated with the differentiated muscle fibers and remain quiescent in the absence of muscle damage. In response to an injury, the quiescent satellite cell is activated by soluble factors, including FGFs released from injured myofibers. Using immunostaining, we here first show that TRPC1 channels are highly expressed in satellite cells attached to muscle fibers. Since CD34, a traditional stem cell marker, was recently found to be expressed in skeletal muscle satellite cells we labeled living satellite cells in their physiological niche associated with host FDB fibers using anti-CD34-FITC antibody. We then monitored intra-cellular calcium in anti-CD34-FITC labeled satellite cells attached to muscle fibers using the calcium sensitive dye X rhod-1 which has little fluorescence cross talk with FITC. FGF2 increased intracellular calcium in satellite cells, which was antagonized by the TRPC channel blocker SKF 96365. Immunostaining showed that NFATc3 is highly expressed in satellite cells, but not in host FDB fibers. Elevation of intracellular calcium by FGF2 is accompanied by nuclear translocation of NFATc3 and NFATc2 and by an increase in the number of MyoD positive cells per muscle fiber, both of which were attenuated by TRPC blocker SKF 96365. Our results suggest a novel pathway of satellite cell activation where FGF2 enhances calcium influx through a TRPC channel, and the increased cytosolic calcium leads to both NFATc3 and NFATc2 nuclear translocation and enhanced number of MyoD positive satellite cells per muscle fiber.

## Introduction

Muscle satellite cells, the muscle resident stem cells of adult skeletal muscle, are located between the basal lamina and the multinucleated mature muscle fibers, and remain quiescent in the absence of muscle damage. In response to an injury, the quiescent satellite cell is activated by soluble factors, including fibroblast growth factors (FGFs) released from injured myofibers, as well as by possible cessation of release of quiescence maintaining factors from the previously undamaged host or other fibers (Montarras et al., 2013). FGF2 is a strong activator of skeletal muscle satellite cells in response to muscle injury. FGF2, released from injured muscle tissue (Bischoff, 1986), binds to satellite cell FGF receptors and activates complex down stream signaling events that remain poorly understood. The number of satellite cells entering proliferation and expressing MyoD, as well as the overall number of satellite cells subsequently transiting into the myogenic state, is enhanced in the presence of FGF2 (Yablonka-Reuveni et al., 1999; Shefer et al., 2006). It was proposed that the enhanced number of satellite cells undergoing myogenesis in the presence of FGF2 reflects a specific effect of the growth factor in recruiting satellite cells into active myogenesis (Yablonka-Reuveni and Rivera, 1994).

The transient receptor potential canonical (TRPC) family consists of 7 members (TRPC1-7) that share a considerable degree of protein homology (Pedersen et al., 2005). Calcium influx via TRPC1 channels is activated by FGF2 in endothelial cells (Antoniotti et al., 2002). In embryonic rat neural stem cells FGF2 induced calcium elevation due to calcium influx through the TRPC1 channel since it can be attenuated by TRPC blockers Gd^3+^ or SKF96365 (Fiorio Pla et al., 2005). FGF2 induced calcium influx through TRPC1 is involved in self-renewal of embryonic rat neural stem cells. TRPC1 is also a necessary component for store-operated calcium entry in myoblasts undergoing migration and differentiation (Louis et al., 2008). TRPC1 expression is significantly upregulated during myogenesis, especially in the presence of sphingosine 1-phosphate, a bioactive lipid involved in satellite cell biology, further implicating a crucial role for TRPC1 in myoblast differentiation (Formigli et al., 2009). In contrast to satellite cells or myoblasts, in adult FDB and EDL muscle fibers, the main isoform of TRPC is TRPC3 (Lanner et al., 2009), and the entry of calcium through TRPC1 channels plays only minor roles in adult muscle fibers (Zanou et al., 2010).

Nuclear factor of activated T cells (NFAT) are a family of transcription factors with ubiquitous roles in muscle differentiation and adaptation (Friday and Pavlath, 2001; Horsley and Pavlath, 2002). Different NFAT family members may have distinct expression patterns and different roles in cellular physiology. In primary cultured skeletal myoblasts, NFATc3 is the first NFAT member to be translocated to the nucleus during differentiation. Targeted loss of NFATc3 in mice leads to decreased primary myogenesis and reduced muscle mass (Abbott et al., 1998; Kegley et al., 2001). In C2C12 myocytes, NFATc3 is the only NFAT isoform that is activated by calcineurin and translocates to the nucleus, while other NFAT family members are not responsive to calcineurin (Abbott et al., 1998; Delling et al., 2000). NFATc2 plays critical roles in skeletal muscle regeneration after muscle damage (Horsley et al., 2001). Therefore, in this report we studied the translocation of both NFATc3 and NFATc2 in satellite cells in response to FGF2.

Here we examine the role of calcium entry via TRPC on the early steps in the activation of muscle satellite cells maintained in their physiological niche attached to living cultured adult skeletal muscle fibers. We identify the satellite cells on living fibers, and reveal for the first time an FGF2 induced increase in satellite cell calcium concentration, which has functional effects on calcium dependent NFAT signaling and on MyoD expression in muscle fiber resident satellite cells.

## Materials and methods

### Isolation, culturing, and identification of satellite cells on living host FDB fibers

CD1 mice were purchased from Charles River. All mice were housed in a pathogen-free area at the University of Maryland, Baltimore. Mice were killed according to authorized procedures of the Institutional Animal Care and Use Committee, University of Maryland Baltimore, by regulated delivery of compressed CO_2_ overdose followed by cervical dislocation. Single muscle fibers were enzymatically dissociated from flexor digitorum brevis (FDB) muscles of 4–5 week old CD-1 mice using 1 h incubation in 0.2% collagenase (Sigma, type I; not further purified) at 37°C. Single fibers were released by trituration and cultured for about 24 h prior to used for experiments as described previously (Liu et al., 1997). Isolated muscle fibers were cultured on laminin-coated glass coverslips. Fibers were cultured in MEM medium supplemented with 5 μg/ml apo transferrin, 5 μg/ml insulin, and 5 ng/ml sodium selenite (Sigma-Aldrich). Media was replaced every 24 h. Living satellite cells attached to living cultured muscle fibers were identified using FITC conjugated primary anti-CD34 antibody added to the culture medium for 3 h, following by 30 min wash with antibody free medium. For MyoD immunostain and NFAT translocation experiments, the muscle fiber cultures were plated after collagenase digestion and cultured overnight in MEM with or without FGF2 before fixation. For calcium recording experiments, the muscle fiber cultures were kept in MEM overnight.

### Immunofluorescence histochemistry of fixed muscle fibers

Muscle cell cultures were fixed with 4% paraformaldehyde at room temperature for 20 min and rinsed three times with PBS. The cultures were then permeabilized with 1% Triton X-100 for 20 min. Non-specific binding sites were blocked by incubation with 4% serum from the same species as secondary antibody. Immunostaining was performed by incubating with the primary antibodies indicated in Results at 4°C, followed by overnight incubation with fluorescent conjugated secondary antibodies. The stained satellite cells and host FDB fibers were visualized with an Olympus Fluoview 500 laser scanning confocal microscope. The average fluorescence of pixels within user-specified areas of interest (AOI) in each image was quantified using Image J. The fluorescence values for the AOI of nucleus and the AOI for the cytoplasm of each individual satellite cell was measured and the ratio of nuclear fluorescence/cytoplasm fluorescence was calculated.

### Primary and secondary antibodies

The following primary antibodies were used: anti-laminin (L9393, rabbit IgG, Sigma, 1:200 dilution); anti-Pax-7 (mouse IgG, ascites fluid, Developmental Studies Hybridoma Bank, 1:100 dilution); anti-MyoD (M-318, rabbit IgG, Santa Cruz Biotechnology, 1:100 dilution); anti-NFATc3 (M-75, rabbit IgG, Santa Cruz Biotechnology, 1:100 dilution); anti-NFATc2 (M-300, rabbit IgG, Santa Cruz Biotechnology, 1:100 dilution); anti-CD34-FITC (RAM34, rat IgG, BD Pharmingen, 1:200 dilution); and anti-TRPC1 (T8276, rabbit IgG, Sigma, 1:100 dilution). The following secondary antibodies were used: donkey anti-mouse Alexa Fluor 647, donkey anti-rabbit Alexa Fluor 488, and donkey anti-rabbit Alexa Fluor 488.

### Calcium recording in anti-CD34-FITC labeled satellite cells

Satellite cells on living muscle fibers were stained with FITC labeled anti-CD34 primary antibody by exposing the living fibers to anti-CD34-FITC for 3 h in the tissue culture incubator at 37°C. Culture medium was then changed to antibody-free normal Ringer's solution. X rhod-1 AM (Molecular Probes) in DMSO was added to dishes to give a final concentration of 1 μM X rhod-1 AM in Ringer's solution. After loading for 10 min, cultures were rinsed with X rhod-1AM free Ringer's solution and equilibrated for 30 min before recording. X rhod-1 was excited with laser of 543 nm and emission signal was collected above 560 nm. FITC was excited at 488 nm and emission signal was recorded through a barrier filter of 505–525 nm. Calcium recording was carried out at room temperature, 21–23°C. The fluorescence was quantitated using Image J. The absolute value of fluorescence of each time point was individually divided (normalized) by the fluorescence value at time = 0 min to obtain the relative change in fluorescence at each time point. All fluorescence measurements (F/F0) are relative to the fluorescence (F0) at a time before FGF2 was added.

All fluorescence images of living or fixed preparations, including the Ca^2+^ images, were obtained by confocal microscope imaging. In contrast, the non-fluorescent transmitted light images, are a result of light scattering, which includes a much larger depth of focus and thus shows structural features, including muscle fiber and satellite cell nuclei, at multiple focal planes, which is not the case for the confocal fluorescence images.

### Data analysis and statistics

All values are presented as means ± s.e.m. Statistical significance was tested with One-Way repeated measures ANOVA (Figure 4) or unpaired *t*-test (Figures 5, 7, 8). For all comparisons, the level of statistical significance was set at *P* < 0.05.

## Results

### Satellite cells located between basal lamina and host FDB

**Figure 1:**
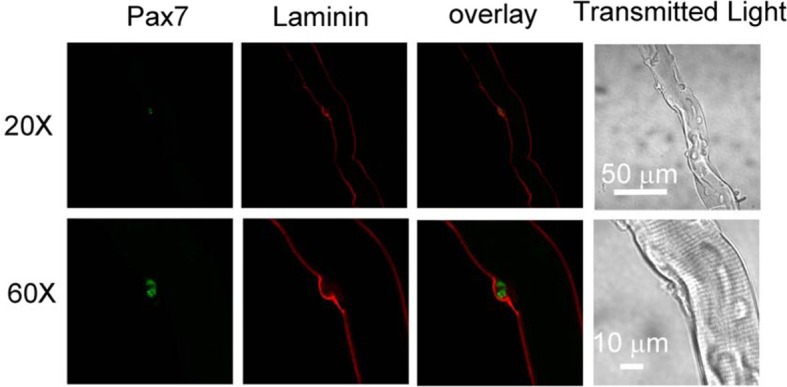
**Satellite cells locate between basal lamina and host FDB in collagenase dissociated FDB cultures.** FDB fiber cultures were fixed with paraformaldehyde and immunostained with anti-laminin anti-Pax7, followed by appropriate secondary antibodies. Satellite cell located between basal lamina and FDB fiber, showing collagenase did not remove lamina and satellite cell from FDB fiber. Images in bottom row are the same fiber as top row but with higher magnification.

We first examined whether satellite cells are attached to host FDB fibers and in their physiological niche (Kuang et al., 2008) after collagenase digestion. FDB fiber cultures were fixed with paraformaldehyde and immunostained with anti-laminin as a marker of basal lamina and anti-Pax7 as a marker of satellite cells. Figure 1 shows that a satellite cell located between basal lamina and FDB fiber, suggesting that collagenase digestion did not strip FDB fiber of its basal lamina and release satellite cells.

### Satellite cells exhibit a high level of expression of TRPC1

**Figure 2:**
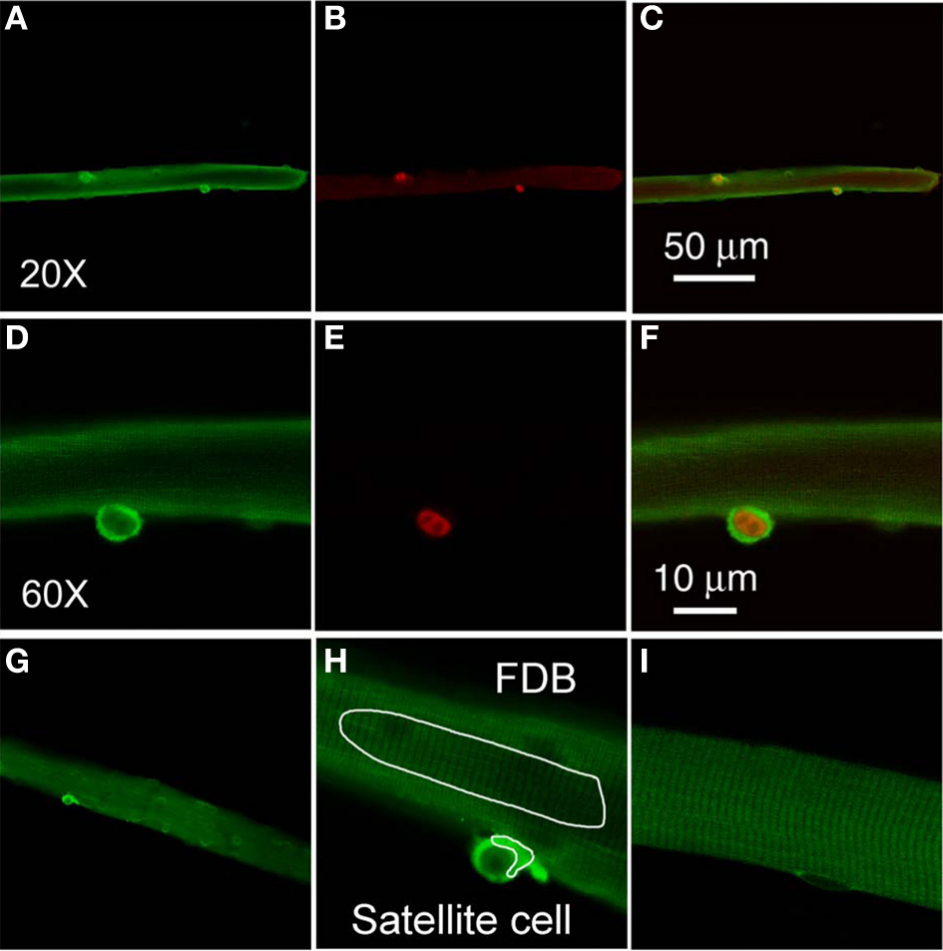
**Satellite cells exhibit a higher level expression of TRPC1.** Freshly isolated FDB fibers were fixed and incubated with anti-TRPC1 and anti-Pax7 antibodies, followed by secondary antibodies conjugated with Alexa Fluor 488 or Alexa Fluor 647, respectively. Satellite cells have stronger stain with anti-TRPC1 antibody than the host FDB fibers **(A,D)**. Satellite cells were identified with anti-Pax7 immunostain **(B,E)**. TRPC1 is localized at both cytoplasm and nuclei of satellite cells **(D)**, whereas Pax7 is localized in the nuclei of satellite cells **(E)**. Overlays are shown in **(C,F)**. **(D,E)** are the same cell as **(A,B)** but with higher magnification. **(G,H)** are images demonstrating how the AOI s are defined in FDB fiber and in satellite cell. **(I)** shows a FDB fiber incubated with only secondary antibody.

Our interest in TRPC in satellite cells was sparked by the report that in neural stem cells FGF2 evokes calcium transients mediated by TRPC1 (Fiorio Pla et al., 2005). To examine whether TRPC1 is differentially expressed in satellite cells compared to the host FDB fibers, we carried out immunofluorescent staining experiments on FDB cultures with satellite cells attached. In freshly dissociated and fixed FDB cultures, immunostaining with anti-TRPC1 followed by secondary antibody shows 1–2 strong positive spots located at the periphery of each FDB fiber, as observed with low power (20×) objective (Figure 2A). The cytoplasm and nuclei of the host FDB fiber exhibited much weaker antibody staining for TRPC1.

The staining of the satellite cells with the anti-TRPC1 is far more intense than that of the FDB fiber cytoplasm in the same confocal image, with a ratio of 3.9 ± 0.4 (satellite cell cytoplasm fluorescence/host fiber cytoplasm fluorescence, data from 22 satellite cells of 19 host FDB fibers from 3 mice). Furthermore, the staining of FDB fibers is indistinguishable from the staining obtained using secondary antibody alone (Figure 2I).

To determine the identity of the TRPC1 stained cells attached to muscle fibers, we next applied anti-Pax7 antibody and corresponding secondary antibody as well as anti-TRPC and its secondary antibody to the muscle fiber cultures. Each of the 38 satellite cells identified by positive Pax7 staining (Figure 2B) in 30 host FDB muscle fibers, was also strongly labeled by anti-TRPC1 antibody, confirming that TRPC1 antibody labels satellite cells.

TRPC1 and Pax-7 are expressed in the same satellite cells. We next used confocal microscopy to resolve the relative distribution of TRPC1 and Pax7 in single satellite cells at the subcellular level (Figures 2D,E; the same muscle fiber as in Figures 2A,B, but at higher magnification, with 60× objective. Figure 2C is overlay of Figures 2A,B. Figure 2F is overlay of Figures 2D,E). TRPC1 is localized in the cytoplasm and nucleus of satellite cells (Figure 2D), with the cytoplasm having much stronger stain than that of nuclei. In contrast, Pax7 is localized to the nucleus of satellite cells as previously reported (Figure 2E; 38 satellite cells of 30 host FDB fibers from 3 mice; Seale et al., 2000). Both markers are present in the same satellite cell, but show different spatial localization within that single cell. Figure 2H shows how AOI s are defined in FDB fiber and in satellite cell. Figure 2G is lower magnification of Figure 2H.

### Identification of living satellite cells on isolated living adult FDB fibers maintained in culture

**Figure 3:**
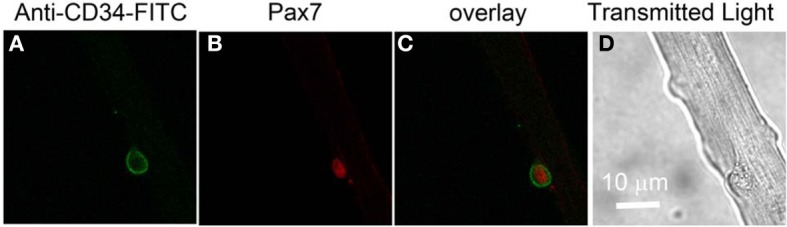
**Identification of satellite cells on living FDB fibers.** Anti-CD34-FITC antibody was added to the cell culture medium and incubated in the cell culture incubator for 3 h. After rinsing with antibody free medium, unbound antibody was washed out and only satellite cells were labeled, with negligible fluorescence appearing on host FDB fibers **(A)**. Culture was then fixed and immunostained with anti-Pax7 primary antibody followed by Alexa Fluor 647 conjugated secondary antibody **(B)**. Overlay is shown in **(C)**. In the transmitted light image **(D)**, the satellite cell is attached to the host FDB fiber.

In order to monitor possible intracellular calcium signals in living satellite cells in their physiological niche, we first developed a method to label satellite cells in living adult FDB fiber cultures using anti-CD34-FITC fluorescent primary antibody (clone RAM34) applied in the culture medium. CD34 is expressed on the cell surface and is a recognized marker for both quiescent and activated satellite cells (Beauchamp et al., 2000; Kuang and Rudnicki, 2008). Anti-CD34-FITC antibody was added to the culture medium bathing overnight cultured muscle fibers at a final dilution of 1:200, without otherwise altering the fiber culture. After 3 h incubation, cultures were rinsed 30 min with antibody free Ringer's solution to wash out the unbound antibody. Only satellite cells were labeled by anti-CD34-FITC, with negligible fluorescence labeling on host FDB fibers (Figure 3A). In the transmitted light image (Figure 3D), the satellite cell is seen attached to the host FBD fiber. After fixation with paraformaldehyde, the muscle cultures were immunostained with anti-Pax7 primary antibody followed by Alexa Fluor 647 conjugated secondary antibody (Figure 3B). Figure 3C shows the co-localization of CD34 and Pax7 stain. The use of fluorescent primary anti-CD34-FITC antibody in the bathing solution thus enables us to locate CD34 expressing satellite cells on host FDB fibers, and to distinguish satellite cells from nuclei of FDB fibers without dissociating satellite cells from the host fibers.

### Effects of FGF2 on cell calcium in satellite cells associated with host muscle fibers

**Figure 4:**
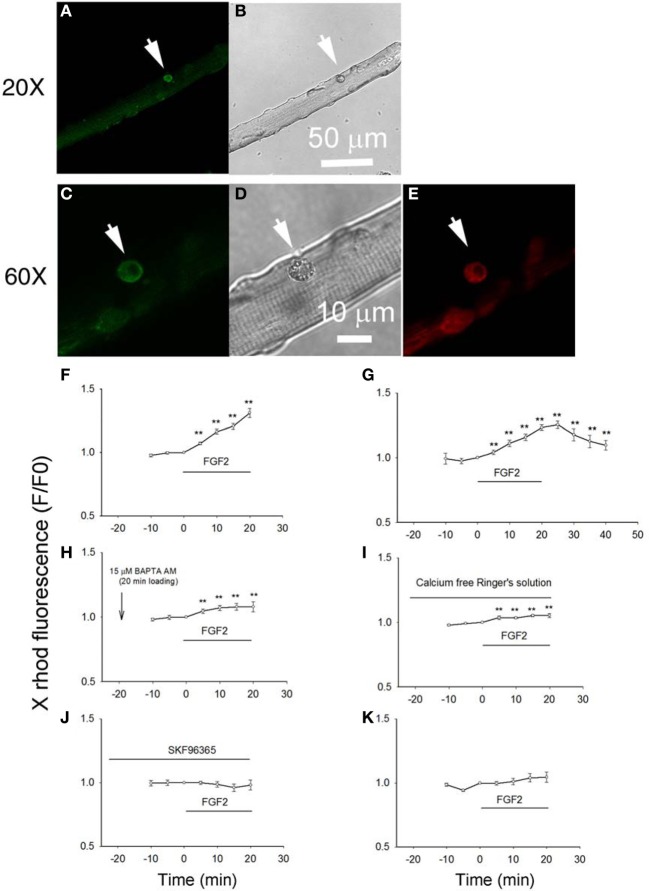
**Studying the effects of FGF2 on cellular calcium in satellite cells associated with host FDB fibers.** Muscle cultures pre-stained with anti-CD34-FITC to identify satellite cells **(A,C)** were then loaded with calcium indicator X rhod-1 AM **(E)**. Transmitted light images are shown in **(B,D)**. Application of FGF2 at 2 ng/ml to the culture triggered a rise of calcium in the satellite cell in the confocal image plane (**F**; 9 satellite cells from 2 mice). Rinse with Ringer's solution without FGF2 reverses the rise in cell calcium (**G**; 9 cells from 2 mice). In cultures pre-loaded with calcium chelator BAPTA AM FGF2 caused little increase in calcium (**H**; 8 cells from 2 mice). In cultures rinsed with calcium free Ringer's solution, the effects of FGF2 are minimal (**I**; 8 cells from 2 mice). Incubation with TRPC blocker SKF 96365 attenuated the effects of FGF2 on cellular calcium (**J**; 8 satellite cells from 2 mice). FGF2 has no effects on cellular calcium of FDB fibers (**K**; 11 FDB fibers from 2 mice). ^**^p < 0.01, compared to 0 min.

Next, we examined the effects of FGF2 on the intracellular calcium concentration of satellite cells in their physiological niche on host FDB fibers. Here we studied the changes in cellular calcium produced by FGF2 and confirmed the involvement of TRPC by using the TRPC channel blocker SKF 93635. We loaded cells, both satellite cells and their host muscle fibers, with the “AM” form of the calcium sensitive dye X rhod-1. After de-esterification of the loaded X rhod-1 AM, we used confocal microscopy to examine calcium signals arising from the satellite cells, but not the host FDB fibers. The living satellite cells in their physiological niche associated with the host FDB fibers were first identified by labeling with anti-CD34-FITC antibody as described above. Calcium indicator X rhod-1 can be excited at 543 nm and has emission at 560 nm and thus has no cross-talk with FITC (Figures 4A,C,E). Application of FGF2 at 2 ng/ml to the culture triggered a steadily increasing rise of calcium in the satellite cell in the confocal image plane (Figure 4F). To study whether the effects of FGF2 on calcium is reversible, muscle culture was rinsed with Ringer's solution after incubated with FGF2 for 20 min. In another 20 min observation period, cell calcium decayed to about 30 percent of peak calcium increase (Figure 4G). If muscle cultures were pre-loaded with BAPTA-AM or the muscle cultures were incubated in calcium free Ringer's plus EGTA, the effects of FGF2 on satellite cell calcium were minimal (Figures 4H,I). Incubation with TRPC blocker SKF 96365 attenuated the effects of FGF2 on cell calcium (Figure 4J), suggesting the calcium entry was through TRPC.

When the host FBD fibers were treated with the same concentration of FGF2, there is no significant change in cellular calcium, suggesting that FGF2 selectively affects cellular calcium of satellite cells (Figure 4K).

### Effects of FGF2 on satellite cell activation and proliferation

As the satellite cells enter the cell cycle, their nuclei become positive for MyoD and for proliferating cell nuclear antigen (PCNA) (Yablonka-Reuveni and Rivera, 1994). MyoD is an extensively studied marker for identifying determined myogenic progenitors (Tapscott, 2005), and is not expressed by quiescent satellite cells in the adult muscle (Yablonka-Reuveni and Rivera, 1994; Cornelison and Wold, 1997). Therefore, the induction of MyoD expression is regarded as an early marker for satellite cell activation. We tested the response of satellite cells to FGF2 in our culture system by counting the MyoD positive (MyoD+) cells associated with each FDB fiber, an approach used by other laboratories to monitor the activation and proliferation of satellite cells (Yablonka-Reuveni and Rivera, 1994; Jones et al., 2005). Muscle fibers were cultured for 24 h under three different conditions (control, with FGF2, or with FGF2 and SKF 96365), and then fixed. Anti-MyoD immunofluorescent staining was used to identify the MyoD+ cells.

**Figure 5:**
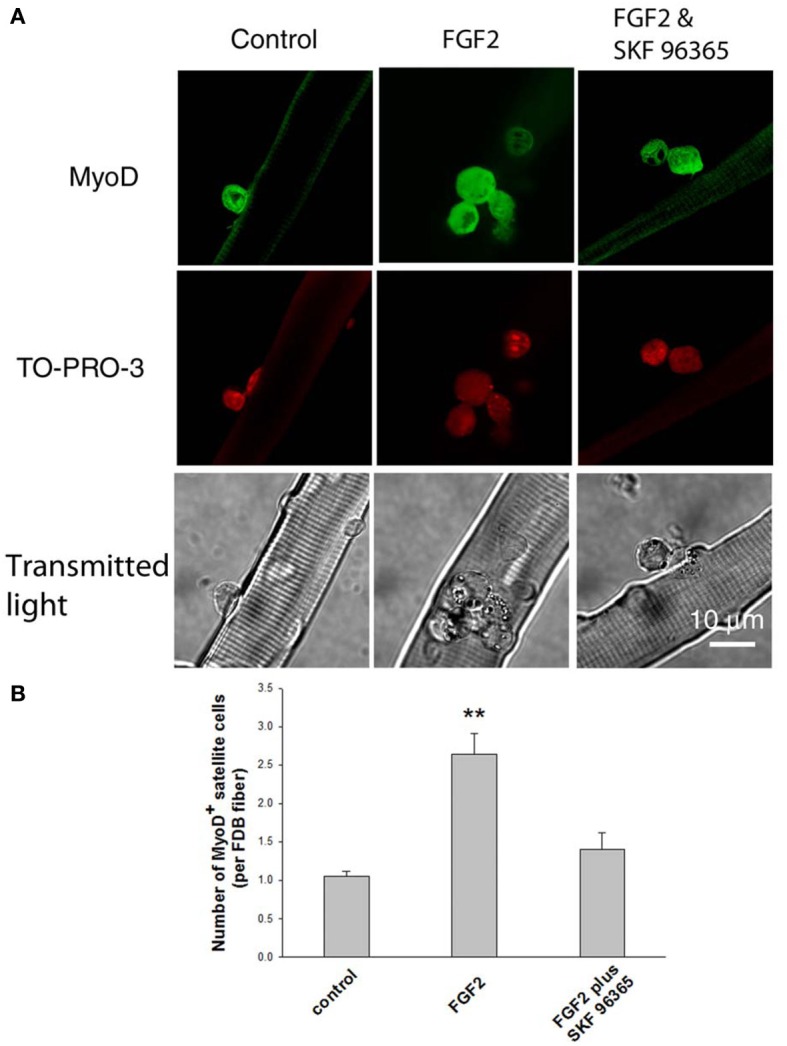
**Effects of FGF2 on satellite cell activation and proliferation.** Freshly isolated FDB fibers associated with satellite cells were cultured for 24 h without FGF2, with FGF2, or with both SKF 96365 and FGF2. FGF2 significantly increased the number of MyoD+ cells associated with FDB fibers (middle column in **A,B**). In cultures treated with both SKF 96365 and FGF2, the induction of MyoD+ cells by FGF2 was partly diminished (right column in **A,B**). Data were from 17 satellite cells of 16 FDB fibers, 37 satellite cells of 14 FDB fibers, and 14 satellite cells of 10 FDB fibers from 3 mice, for left, middle, and right column respectively. ^**^p < 0.01 compared to control.

We found that under control conditions there are 1–2 MyoD+ cells per FDB fiber (Figures 5A,B, left column), similar to the number of satellite cells identified per fiber using anti CD34-FITC on living fibers (above). FGF2 significantly enhanced the number of MyoD+ cells associated with FDB fibers, as previously reported (Yablonka-Reuveni and Rivera, 1994; Yablonka-Reuveni et al., 1999; Jones et al., 2005). The MyoD+ cells were increased to about 2.5 per FDB after 24 h exposure to FGF2 (Figures 5A,B, middle column). Since TRPC blocker SKF 96365 attenuates the calcium transients triggered by FGF2, we next asked the question whether SKF 96365 can block the activation of satellite cells by FGF2. In the presence of 1 μM SKF 96365, the induction of MyoD+ cells by FGF2 was considerably diminished (Figures 5A,B, right column); suggesting the activation of MyoD and satellite cell proliferation by FGF2 is at least partly mediated by TRPC.

### Both NFATc3 and NFATc2 are expressed in the cytoplasm of quiescent satellite cells on cultured FDB fibers

**Figure 6:**
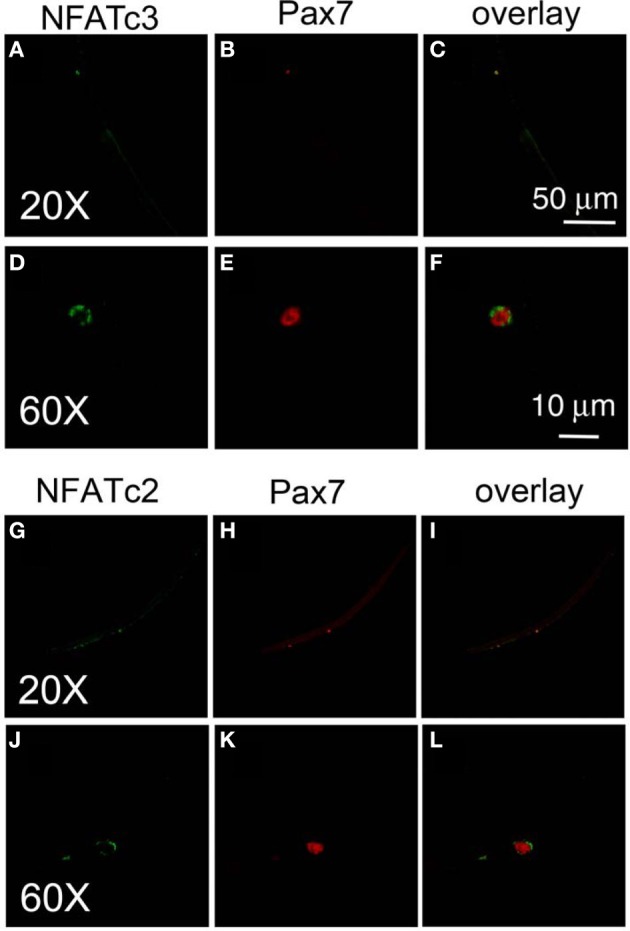
**Both NFATc3 and NFATc2 are expressed in satellite cells.** Freshly isolated FDB fibers and associated satellite cells were fixed and immunostained with anti-NFATc3 and anti-Pax7, or with anti-NFATc2 and anti-Pax7. NFATc3 is expressed in satellite cells, with much higher staining levels than in the adult host FDB fibers **(A,D)**. Detailed analysis shows that NFATc3 localizes in the cytoplasm not in the nucleus in the non-activated satellite cells in contrast to Pax7 **(B,E)**. NFATc2 is expressed in satellite cells, localized at cytoplasm **(G,J)** in contrast to Pax7 which is in the nucleus **(H,K)**. **(D–F)** are the same cell as **(A–C)** but at higher magnification. **(J–L)** are the same cell as **(G–I)** but with higher magnification.

To further study the down stream target of calcium influx through TRPC1 in satellite cell activation, we next examined the calcium dependent subcellular distribution of NFATc3 and NFATc2 in satellite cells on muscle fibers exposed to various conditions and then fixed and immunostained using anti-NFATc3 or anti-NFATc2 and corresponding secondary antibody. We chose to examine NFATc3 because NFATc3 is the NFAT isoform playing important roles in early myogenesis (Abbott et al., 1998). Figure 6 shows that NFATc3 is expressed in quiescent satellite cells at a much higher staining level than in the resting adult host FDB fibers (with a ratio of 7.7 ± 0.7; satellite cell cytoplasmic fluorescence/FDB fiber cytoplasmic fluorescence; 13 satellite cells on 13 FDB fibers from 2 mice). Closer examination shows that NFATc3 localizes to the cytoplasm, but not to the nucleus in non-activated satellite cells (Figures 6A,D). This is in contrast to Pax7, which is concentrated in the nuclei of quiescent satellite cells (Figures 6B,E). Similar to NFATc3, NFATc2 is also expressed in satellite cells and localized to the cytoplasm non-activated satellite cells (Figures 6G,J).

### FGF2 causes NFATc3 and NFATc2 nuclear translocation, which can be blocked by SKF96365

**Figure 7:**
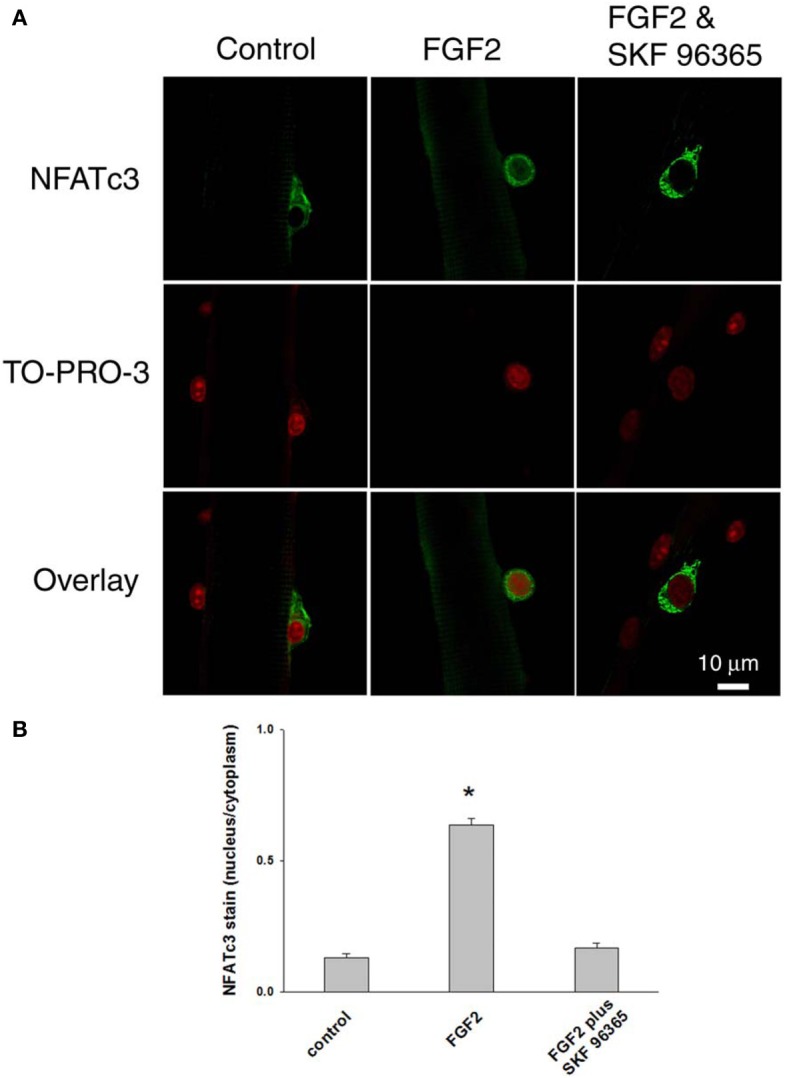
**Documenting NFATc3 activation by imaging NFATc3 translocation.** NFATc3 is located in the cytoplasm and not in the nucleus in quiescent satellite cells in the absence of FGF2 in (**A,B**; left column). In FGF2 treated satellite cells, NFATc3 is located in both cytoplasm and nucleus (**A,B**; middle column). TRPC blocker SKF 96365 antagonized the NFATc3 nuclear translocation triggered by FGF2 (**A,B**; right column). Nucleus/cytoplasm ratio of NFATc3 was calculated by quantification of nuclear and cytoplasmic fluorescence of satellite cells immunostained with anti-NFATc3 antibody **(B)**. The nuclei of fixed muscle cultures were stained with TO-PRO-3 (Invitrogen, Molecular Probes; 1:1,000 in PBS) for 10 min at room temperature, followed by rinsing with PBS before imaged. ^*^p < 0.05 compared to control.

After we established that NFATc3 and NFATc2 are localized in the cytoplasm and not in the nucleus in quiescent satellite cells in the absence of FGF2, we next investigated whether FGF2, which is capable of activating satellite cell proliferation, would also result in nuclear translocation of NFATc3 and NFATc2. Satellite cells and the host muscle fibers were cultured for 12 h with or without FGF2, then fixed and stained with anti-NFATc3 or anti-NFATc2 antibodies. In stark contrast to the situation without FGF2, in FGF2 treated satellite cells, NFATc3 is located in both cytoplasm and nucleus (Figures 7A,B, middle column). TO-PRO-3 was used to label the nuclei. Since TRPC blocker SKF 96365 is able to partially inhibit the effects of FGF2 on satellite cell proliferation monitored with MyoD immunostain (Figure 5), we asked the question whether SKF 96365 can block the effects of FGF2 on NFATc3 nuclear translocation. Muscle cultures were incubated first with SKF 96365 at 1 μM. Then the muscle culture was treated with FGF2 for 12 h with SKF 96365 in the medium. SKF 96365 antagonized the NFATc3 nuclear translocation by FGF2 (Figures 7A,B, right column).

The subcellular distribution of NFATc3 was further quantified. As summarized in Figure 7B, in cultures without FGF2, the nucleus/cytoplasm mean pixel fluorescence ratio for anti NFATc3 antibody staining is 0.132 ± 0.013 (mean ± SE, data from 12 satellite cells of 12 FDB fibers from 3 mice). In the FGF2 treated cultures, the ratio is 0.635 ± 0.026 (data from 22 satellite cells of 10 FDB fibers from 3 mice, *p* < 0.05 compared to control), demonstrating a clear increase in nuclear NFATc3 compared to the cultures without FGF2. In the group of cultures which were exposed to FGF2 after treatment with SKF 96365, the ratio is 0.167 ± 0.020 (data from 9 satellite cells of 9 FDB fibers from 3 mice; *p* < 0.05, compared to FGF2 alone), showing that the TRPC blocker antagonized the nuclear translocation of NFATc3 by FGF2. Thus, NFATc3 nuclear/cytoplasmic antibody staining exhibits an FGF2 dependent increase, which is largely suppressed in the presence of TRPC blocker SKF 96365.

**Figure 8:**
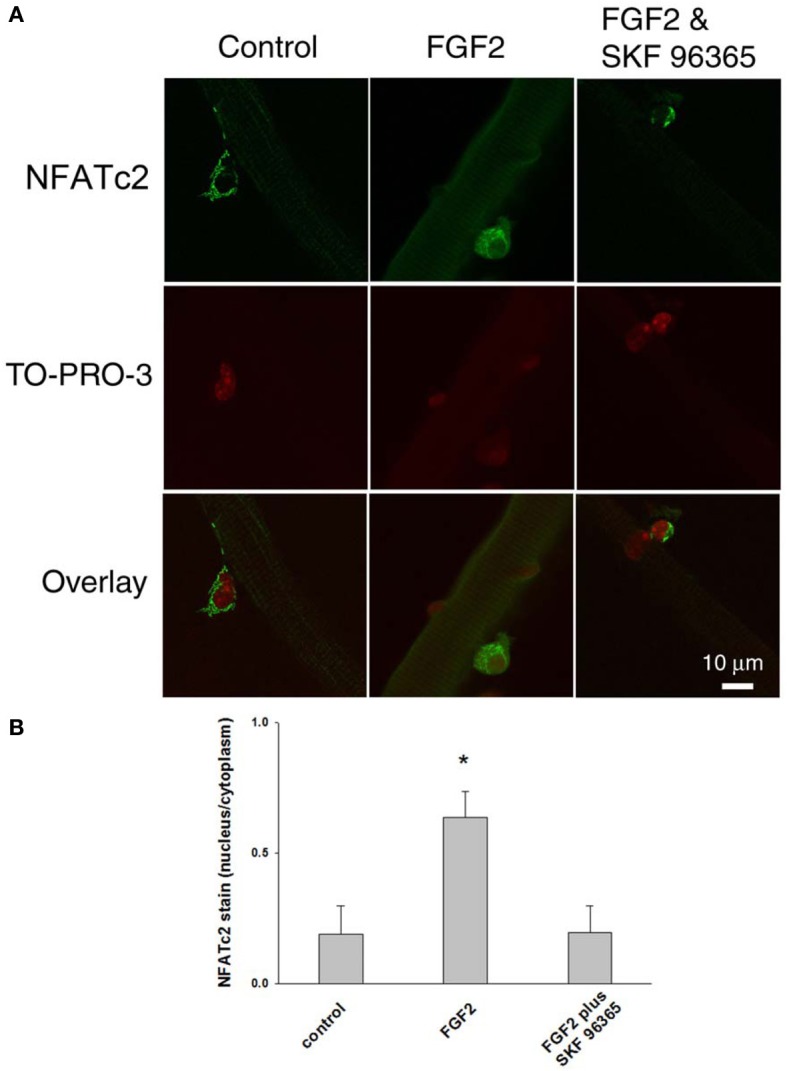
**FGF2 caused NFATc2 nuclear translocation.** NFATc2 is not in the nucleus in the absence of FGF2 (**A,B**; left column). In the presence of FGF2, NFATc2 is located in both nucleus and cytoplasm (**A,B**; middle column). TRPC blocker SKF 96365 antagonized the NFATc2 nuclear translocation caused by FGF2 (**A,B**; right column). Nucleus/cytoplasm ratio of NFATc2 was calculated by quantification of nuclear and cytoplasmic fluorescence of satellite cells immunostained with anti-NFATc2 antibody **(B)**. ^*^p < 0.05 compared to control.

We further examined the response of NFATc2 to FGF2. As shown in Figure 8, FGF2 caused nuclear translocation of NFATc2. The nucleus/cytoplasm mean pixel fluorescence ratio for anti-NFATc2 antibody staining is 0.189 ± 0.110 (mean ± SE, data from 11 satellite cells of 11 FDB fibers from 3 mice) in absence of FGF2. In the FGF2 treated cultures, the ratio is 0.637 ± 0.100 (data from 14 satellite cells of 10 FDB fibers from 3 mice, *p* < 0.05 compared to control), demonstrating a significant increase in nuclear NFATc2 compared to the cultures without FGF2. In the group of cultures which were exposed to FGF2 in the presence of SKF 96365, the ratio is 0.197 ± 0.100 (data from 11 satellite cells of 11 FDB fibers from 3 mice), suggesting that the TRPC blocker antagonized the nuclear translocation of NFATc2 by FGF2. In summary our results show that both NFATc2 and NFATc3 nuclear/cytoplasmic antibody stain exhibit an FGF2 dependent increase, which is blocked in the presence of TRPC blocker SKF 96365.

## Discussion

The major new findings in this report are that TRPC1 is highly expressed in satellite cells, and that FGF2 triggers elevated cell calcium by activating TRPC, which leads to NFATc3 and NFATc2 nuclear translocation and increased expression of MyoD. These effects of FGF2 on satellite cell calcium and related responses were studied in satellite cells maintained in their physiological niche on the surface of isolated adult muscle fibers. Our procedure follows the general muscle fiber enzymatic dissociation protocol developed by Bischoff (1986) to maintain the satellite cells within the basement membrane after muscle fiber isolation. Although Bischoff (1986) fractionated the enzyme prior to use on muscle fibers, others have found that satellite cells are maintained on muscle fibers after dissociation with non-fractionated commercially available collagenase preparations (Yablonka-Reuveni and Rivera, 1994; Yablonka-Reuveni et al., 1999). Here we present evidence showing that basal lamina is not striped by collagenase digestion.

### Identifying quiescent satellite cells on isolated adult muscle fibers

An important aspect of the present study is the development of a technique to identify quiescent satellite cells in their physiological niche on the surface of dissociated adult FDB fibers (Kuang et al., 2008). In general, Pax7 is probably the most useful maker for identifying quiescent satellite cells due to the availability of a good antibody (Seale et al., 2000; Shefer et al., 2006). However, since Pax7 is a nuclear transcriptional factor, and is not present on the cell surface, identifying satellite cells with anti-Pax7 antibody cannot be applied to living muscle fibers, but only can be used with fixed and permeabilized muscle tissue. In living muscle fiber cultures, CD34 is a better choice as a marker of satellite cells since it is on the cell external surface and fluorophore conjugated primary anti CD34 antibodies are available. CD34 is a highly glycosylated trans membrane sialomucin. It was first found in hematopoietic stem and progenitor cells (Krause et al., 1996) and in blood vessel endothelium (Baumhueter et al., 1994). By combining CD34 with the two other satellite cell markers Myf5 and M-cadherin, Beauchamp et al. (2000) reported that CD34 is expressed in most, but not all, quiescent satellite cells associated with isolated single muscle fibers. Thus, our CD34 antibody staining may miss some of the quiescent satellite cells present on our cultured fibers. CD34 is also found in satellite cells activated by culture in serum containing media, and thus positive for MyoD (Beauchamp et al., 2000). Since the discovery of CD34 as cell surface marker of satellite cells, several studies have used CD34 as a positive selection marker to enrich muscle stem cells in fluorescence-activated cell sorting for stem cell transplantation (Montarras et al., 2005; Sacco et al., 2008), but it has not been used previously to identify living quiescent or activated satellite cells on living isolated muscle fibers.

### FGF2 activation of TRPC channels

It is presently not clear as to how the activation of FGF receptor might enhance the calcium influx through TRPC. However, it has been reported that in rat neural stem cells TRPC1 is embedded in a protein complex, which can also include FGF receptors (Fiorio Pla et al., 2005). Therefore it is plausible that direct interaction between active FGF receptors and TRPC channels leads to the augmentation of calcium influx. This is consistent with the report that TRPC1 responds to general stimuli, not only depletion of intracellular calcium stores, but also receptor activation. In addition to TRPC, several members of the TRPV and TRPM subfamilies of TRP channels are expressed in skeletal muscle (Kunert-Keil et al., 2006; Krüger et al., 2008). In adult skeletal muscle TRPC3 channels are involved in insulin mediated glucose uptake (Lanner et al., 2009). Activation of calcium signaling through TRPV1 plays important roles in skeletal muscle hypertrophy (Ito et al., 2013). Whether these isoforms of TRP are expressed in satellite cells and play functional roles needs further study.

### NFAT in satellite cells

Individual NFAT family members may have distinct expression patterns and different roles at the different stages of skeletal muscle development (Horsley and Pavlath, 2002). In cultured skeletal myoblasts, NFATc3 is the first NFAT member to be translocated to the nucleus during differentiation. Targeted loss of NFATc3 in mice leads to decreased primary myogenesis and reduced muscle mass (Abbott et al., 1998; Kegley et al., 2001). In C2C12 myocytes, NFATc3 is the NFAT isoform that can be activated by calcineurin and translocates to the nucleus, while other NFAT family members do not respond to calcineurin (Abbott et al., 1998; Delling et al., 2000). These observations suggest that NFATc3, rather than other NFAT members, is the main NFAT protein involved in skeletal muscle myogenesis during the early stage of muscle formation. NFATc2 is another important NFAT isoform playing critical roles satellite cell activation. It has been reported that NFATc2 null mice have impaired regeneration after skeletal muscle damage (Horsley et al., 2001), suggesting that NFATc2 is involved in satellite cell activation after muscle damage. However, since NFATc2 null mice have impaired muscle regeneration, whereas NFATc3 null mice have normal regeneration after muscle fiber damage, a role for NFATc3 in satellite cell activation after muscle damage in adult muscle is questioned (Horsley et al., 2001).

In summary, in this report we describe a method to label living satellite cells in their physiological niche with anti-CD34-FITC. We found that FGF2 increases cytosolic calcium through TRPC, which leads to both NFATc3 and NFATc2 nuclear translocation and an increase in MyoD+ satellite cells. More studies are needed to examine the potential functional roles of other TRP channels in the activation of NFATs during the activation of satellite cells. The coupling between FGF receptor activation and calcium influx through TRPC also warrant further investigation.

### Conflict of interest statement

The authors declare that the research was conducted in the absence of any commercial or financial relationships that could be construed as a potential conflict of interest. The Associate Editor, Maegen A. Ackermann, declare that, despite being affiliated with the same institution as the author, Yewei Lie, the review process was handled objectively and no conflict of interest exists.

## References

[B1] Abbott K. L. Friday B. B. Thaloor D. Murphy T. J. Pavlath G. K. (1998). Activation and cellular localization of the cyclosporine A-sensitive transcription factor NF-AT in skeletal muscle cells. Mol. Biol. Cell. 9, 2905–2916 10.1091/mbc.9.10.2905 9763451 PMC25565

[B2] Antoniotti S. Lovisolo D. Fiorio Pla A. Munaron L. (2002). Expression and functional role of bTRPC1 channels in native endothelial cells. FEBS Lett. 510, 189–195 10.1016/S0014-5793(01)03256-2 11801252

[B3] Baumhueter S. Dybdal N. Kyle C. Lasky L. A. (1994). Global vascular expression of murine CD34, a sialomucin-like endothelial ligand for L-selectin. Blood 84, 2554–2565 7522633

[B4] Beauchamp J. R. Heslop L. Yu D. S. Tajbakhsh S. Kelly R. G. Wernig A. (2000). Expression of CD34 and Myf5 defines the majority of quiescent adult skeletal muscle satellite cells. J. Cell Biol. 151, 1221–1234 10.1083/jcb.151.6.1221 11121437 PMC2190588

[B5] Bischoff R. (1986). Proliferation of muscle satellite cells on intact myofibers in culture. Dev. Biol. 115, 129–139 10.1016/0012-1606(86)90234-4 3516758

[B6] Cornelison D. D. Wold B. J. (1997). Single-cell analysis of regulatory gene expression in quiescent and activated mouse skeletal muscle satellite cells. Dev. Biol. 191, 270–283 10.1006/dbio.1997.8721 9398440

[B7] Delling U. Tureckova J. Lim H. W. De Windt L. J. Rotwein P. Molkentin J. D. (2000). A calcineurin-NFATc3-dependent pathway regulates skeletal muscle differentiation and slow myosin heavy-chain expression. Mol Cell Biol. 20, 6600–6611 10.1128/MCB.20.17.6600-6611.2000 10938134 PMC86143

[B8] Fiorio Pla A. Maric D. Brazer S. C. Giacobini P. Liu X. Chang Y. H. (2005). Canonical transient receptor potential 1 plays a role in basic fibroblast growth factor (bFGF)/FGF receptor-1-induced Ca^2+^ entry and embryonic rat neural stem cell proliferation. J. Neurosci. 25, 2687–2701 10.1523/JNEUROSCI.0951-04.2005 15758179 PMC6725156

[B9] Formigli L. Sassoli C. Squecco R. Bini F. Martinesi M. Chellini F. (2009). Regulation of transient receptor potential canonical channel 1 (TRPC1) by sphingosine 1-phosphate in C2C12 myoblasts and its relevance for a role of mechanotransduction in skeletal muscle differentiation. J. Cell Sci. 122(Pt 9), 1322–1333 10.1242/jcs.035402 19351713

[B10] Friday B. B. Pavlath G. K. (2001). A calcineurin- and NFAT-dependent pathway regulates Myf5 gene expression in skeletal muscle reserve cells. J. Cell Sci. 114(Pt 2), 303–310 11148132 10.1242/jcs.114.2.303

[B11] Horsley V. Friday B. B. Matteson S. Kegley K. M. Gephart J. Pavlath G. K. (2001). Regulation of the growth of multinucleated muscle cells by an NFATC2-dependent pathway. J. Cell Biol. 153, 329–338 10.1083/jcb.153.2.329 11309414 PMC2169453

[B12] Horsley V. Pavlath G. K. (2002). NFAT: ubiquitous regulator of cell differentiation and adaptation. J. Cell Biol. 156, 771–774 10.1083/jcb.200111073 11877454 PMC2173310

[B13] Ito N. Ruegg U. T. Kudo A. Miyagoe-Suzuki Y. Takeda S. (2013). Activation of calcium signaling through Trpv1 by nNOS and peroxynitrite as a key trigger of skeletal muscle hypertrophy. Nat. Med. 19, 101–106 10.1038/nm.3019 23202294

[B14] Jones N. C. Tyner K. J. Nibarger L. Stanley H. M. Cornelison D. D. Fedorov Y. V. (2005). The p38alpha/beta MAPK functions as a molecular switch to activate the quiescent satellite cell. J. Cell Biol. 169, 105–116 10.1083/jcb.200408066 15824134 PMC2171902

[B15] Kegley K. M. Gephart J. Warren G. L. Pavlath G. K. (2001). Altered primary myogenesis in NFATC3(-/-) mice leads to decreased muscle size in the adult. Dev. Biol. 232, 115–126 10.1006/dbio.2001.0179 11254352

[B16] Krause D. S. Fackler M. J. Civin C. I. May W. S. (1996). CD34, structure, biology, and clinical utility. Blood 87, 1–13 8547630

[B17] Krüger J. Kunert-Keil C. Bisping F. Brinkmeier H. (2008). Transient receptor potential cation channels in normal and dystrophic mdx muscle. Neuromuscul. Disord. 18, 501–513 10.1016/j.nmd.2008.04.003 18504127

[B18] Kuang S. Gillespie M. A. Rudnicki M. A. (2008). Niche regulation of muscle satellite cell self-renewal and differentiation. Cell Stem Cell 2, 22–31 10.1016/j.stem.2007.12.012 18371418

[B19] Kuang S. Rudnicki M. A. (2008). The emerging biology of satellite cells and their therapeutic potential. Trends Mol. Med. 14, 82–91 10.1016/j.molmed.2007.12.004 18218339

[B20] Kunert-Keil C. Bisping F. Krüger J. Brinkmeier H. (2006). Tissue-specific expression of TRP channel genes in the mouse and its variation in three different mouse strains. BMC Genomics 7:159 10.1186/1471-2164-7-159 16787531 PMC1557673

[B21] Lanner J. T. Bruton J. D. Assefaw-Redda Y. Andronache Z. Zhang S. J. Severa D. (2009). Knockdown of TRPC3 with siRNA coupled to carbon nanotubes results in decreased insulin-mediated glucose uptake in adult skeletal muscle cells. FASEB J. 23, 1728–1738 10.1096/fj.08-116814 19141536

[B22] Liu Y. Carroll S. L. Klein M. G. Schneider M. F. (1997). Calcium transients and calcium homeostasis in adult mouse fast-twitch skeletal muscle fibers in culture. Am. J. Physiol. 272, C1919–C1927 9227421 10.1152/ajpcell.1997.272.6.C1919

[B23] Louis M. Zanou N. Van Schoor M. Gailly P. (2008). TRPC1 regulates skeletal myoblast migration and differentiation. J. Cell Sci. 121(Pt 23), 3951–3959 10.1242/jcs.037218 19001499

[B24] Montarras D. Morgan J. Collins C. Relaix F. Zaffran S. Cumano A. (2005). Direct isolation of satellite cells for skeletal muscle regeneration. Science 309, 2064–2067 10.1126/science.1114758 16141372

[B33] Montarras D. L'honoré A. Buckingham M. (2013). Lying low but ready for action: the quiescent muscle satellite cell. FEBS J. 280, 4036–4050 10.1111/febs.12372 23735050

[B25] Pedersen S. F. Owsianik G. Nilius B. (2005). TRP channels: an overview. Cell Calcium 38, 233–252 10.1016/j.ceca.2005.06.028 16098585

[B26] Sacco A. Doyonnas R. Kraft P. Vitorovic S. Blau H. M. (2008). Self-renewal and expansion of single transplanted muscle stem cells. Nature 456, 502–506 10.1038/nature07384 18806774 PMC2919355

[B27] Seale P. Sabourin L. A. Girgis-Gabardo A. Mansouri A. Gruss P. Rudnicki M. A. (2000). Pax7 is required for the specification of myogenic satellite cells. Cell 102, 777–786 10.1016/S0092-8674(00)00066-0 11030621

[B28] Shefer G. Van de Mark D. P. Richardson J. B. Yablonka-Reuveni Z. (2006). Satellite-cell pool size does matter: defining the myogenic potency of aging skeletal muscle. Dev. Biol. 294, 50–66 10.1016/j.ydbio.2006.02.022 16554047 PMC2710453

[B29] Tapscott S. J. (2005). The circuitry of a master switch: Myod and the regulation of skeletal muscle gene transcription. Development 132, 2685–2695 10.1242/dev.01874 15930108

[B30] Yablonka-Reuveni Z. Rivera A. J. (1994). Temporal expression of regulatory and structural muscle proteins during myogenesis of satellite cells on isolated adult rat fibers. Dev. Biol. 164, 588–603 10.1006/dbio.1994.1226 7913900 PMC4128087

[B31] Yablonka-Reuveni Z. Seger R. Rivera A. J. (1999). Fibroblast growth factor promotes recruitment of skeletal muscle satellite cells in young and old rats. J. Histochem. Cytochem. 47, 23–42 10.1177/002215549904700104 9857210

[B32] Zanou N. Shapovalov G. Louis M. Tajeddine N. Gallo C. Van Schoor M. (2010). Role of TRPC1 channel in skeletal muscle function. Am. J. Physiol. Cell Physiol. 298, C149–C162 10.1152/ajpcell.00241.2009 19846750 PMC2806157

